# Supplementary material for: Content-rich biological network constructed by mining PubMed abstracts
Source: BMC Bioinformatics. 2004 Oct 8;5:147. doi: 10.1186/1471-2105-5-147 (PMC528731; doi:10.1186/1471-2105-5-147)
Supplement: Additional File 2 — The original results of the above study (non-essential files are deleted to keep the file size under the limit set by BMC bioinformatics). [file 1471-2105-5-147-S2.bz2 › chilibotAdditionalFile2/dip10/323ID9824158E3491/html/E3L_PRKR.html]

 


 **E3L** and **PRKR** 
  
Found 25 abstracts in PubMed, retrieved 10.  
 

 What does Google say? 
 PDF only 
| .edu only 

---

**Interactive relationship** (e.g. stimulation, inhibition, etc)

**Inhibitory relationship**- The VV  **E3L**  protein is a potent inhibitor of PKR  [ **PRKR** ] .  Ref: 11333890 J Virol, 2001
- **E3L**  and K3L markedly reduced the activation of the double stranded RNA dependent protein kinase, PKR  [ **PRKR** ] , and led to a significant reduction in apoptosis in HeLa cells.  Ref: 11878896 Virology, 2001
- The vaccinia virus VV  **E3L**  gene encodes a dsRNA binding protein that inhibits activation of the IFN induced, dsRNA dependent protein kinase, PKR  [ **PRKR** ] , the 2 5A synthetases RNase L system and other dsRNA dependent pathways, thus leading to efficient VV replication.  Ref: 12466958 Oncogene, 2002
- However, unlike most poxviruses, MCV lacks a homologue to the  **E3L**  and K3L proteins of vaccinia virus, which are involved in the control of the key antiviral and pro apoptotic dsRNA dependent protein kinase, PKR  [ **PRKR** ] .  Ref: 11714980 J Gen Virol, 2001
**Neutral relationship**- To explore how  **E3L**  enables vaccinia virus to evade the interferon system, cells and mice deficient in the principal interferon regulated antiviral enzymes, PKR  [ **PRKR** ]  and RNase L, were infected with wild type vaccinia virus and strains of vaccinia virus from which  **E3L**  had been deleted  **E3L**  deleted strains.  Ref: 11967338 J Virol, 2002
- IFN pretreated cells infected with VV in which the  **E3L**  locus was replaced with the NS5A gene VVNS5A displayed diminished phosphorylation of PKR  [ **PRKR** ]  and eIF2alpha in a transient manner.  Ref: 11333890 J Virol, 2001
- Accordingly, infection of IFN pretreated HeLa S3 cells with an  **E3L**  deficient VV VVDeltaE3L resulted in increased phosphorylation levels of both PKR  [ **PRKR** ]  and eIF2alpha.  Ref: 11333890 J Virol, 2001

**Non-interactive relationship** (e.g. studied together, co-existance, homology, etc.)

- Vaccinia virus was able to overcome these cellular variations by regulating PKR  [ **PRKR** ]  activity through the synthesis of either  **E3L**  or K3L.  Ref: 12167348 Virology, 2002
- While wild type virus was unaffected by RNase L and PKR  [ **PRKR** ] , virus lacking  **E3L**  replicated only in the deficient cells.  Ref: 11967338 J Virol, 2002
- Nevertheless,  **E3L**  deleted virus failed to replicate to high titers or to cause significant morbidity or mortality in triply deficient mice lacking RNase L, PKR  [ **PRKR** ] , and Mx1.  Ref: 11967338 J Virol, 2002
- Nonphosphorylatable mutant eIF2alpha, knockout of PKR  [ **PRKR** ]  and PKR  [ **PRKR** ]  inhibitors 2 aminopurine, transdominant negative PKR  [ **PRKR** ] , or vaccinia  **E3L**  correspondingly enhances translation of IFN gamma mRNA.  Ref: 11832212 Cell, 2002
- The role of the PKR  [ **PRKR** ]  inhibitory genes,  **E3L**  and K3L, in determining vaccinia virus host range.  Ref: 12167348 Virology, 2002
- Data obtained from infection of epithelial cells with a vaccinia virus lacking the PKR  [ **PRKR** ]  inhibitory polypeptide,  **E3L** , revealed that PKR  [ **PRKR** ]  activation was essential for TNF alpha but not for IL 1 beta expression.  Ref: 12055262 J Immunol, 2002
